# Motoric cognitive risk syndrome as a predictor of incident disability: A 7 year follow-up study

**DOI:** 10.3389/fnagi.2022.972843

**Published:** 2022-09-08

**Authors:** Anying Bai, Weimin Bai, Hepeng Ju, Weihao Xu, Zhanyi Lin

**Affiliations:** ^1^Department of Epidemiology and Biostatistics, School of Public Health, Chinese Academy of Medical Sciences and Peking Union Medical College, Beijing, China; ^2^Department of Emergency, Henan Provincial People's Hospital, People's Hospital of Zhengzhou University, People's Hospital of Henan University, Zhengzhou, China; ^3^Center for Disease Control and Prevention of Southern Theatre Command, Guangzhou, China; ^4^Department of Geriatrics, Guangdong Provincial Geriatrics Institute, Guangdong Provincial People's Hospital, Guangdong Academy of Medical Sciences, Guangzhou, China

**Keywords:** motoric cognitive risk syndrome, ADL disability, IADL disability, older adult, cohort study

## Abstract

**Background:**

Though motoric cognitive risk syndrome (MCR) share risk factors with disability, whether it predict disability remains understudied.

**Objectives:**

This study aims to examine the association between MCR and incident disability.

**Design:**

Longitudinal study.

**Methods:**

MCR was defined as subjective cognitive complaints and objective slow gait speed. Two subtypes of MCR were defined by whether memory impairment (MI) was also present, MCR-MI and MCR-non-MI. Incident activities of daily living (ADL) disability and instrumental activities of daily living (IADL) disability were outcome measures. Multiple logistic regression analysis was used to assess the independent effect of MCR at baseline on the odds of ADL/IADL disability at a 7 year follow-up.

**Results:**

Among the subjects who were not disabled at baseline and followed for 7 years, 34.66% reported incident ADL disability, and 31.64% reported incident IADL disability. Compared with participants without MCR at baseline, those with MCR had 58% increased odds of incident ADL disability (OR=1.58, 95% CI: 1.19–2.09) and 46% increased odds of incident IADL disability (OR=1.46, 95% CI: 1.13–1.88) after 7 years. MCR-non-MI was associated with a 56.63% increased risk of ADL disability and a 34.73% increased risk of IADL disability. MCR-MI was associated with an even higher risk of IADL disability (OR = 2.14, 95% CI: 1.18–3.88).

**Conclusions:**

MCR is an independent risk factor for both incident ADL and IADL disability. MCR-MI predicts a higher risk for disability than MCR-non-MI. Early identification of MCR among older adult is recommended and may decrease future risk of disability.

## Key points

- MCR share risk factors with disability.
- MCR is an independent risk factor for both incident ADL and IADL disability.
- MCR participants are characterized as MCR-MI if having memory impairment or MCR-non-MI if there was no memory impairment.
- MCR-MI predicts a higher risk for IADL disability than MCR-non-MI.

## Introduction

The unprecedented upward shift in life expectancy has increased the aging-related challenges faced by middle-income countries (Kontis et al., 2017). According to the latest data from the seventh population census in China, older adults (≥60 years) make up 18.7% (264 million) of the total population (Tu et al., 2022). Older adults have decreased functional capacity (e.g., sensory, cognitive, and physical), which poses significant medical and socioeconomic challenges across societies. In tandem with a world population experiencing a pervasive process of aging, the recently described motoric cognitive risk (MCR) syndrome is characterized by cognitive complaints and slow gait (Verghese et al., 2014), identifying non-demented older individuals at high risk for transitioning to dementia. The prevalence of MCR is in the range 2–18%, and a multi-country study showed that the worldwide prevalence of MCR was estimated at 9.7% among 26,082 older adults (age range: 60–114 years) (Merchant et al., 2021).

Older adults are vulnerable to adverse events, such as disability, dependency, falls, or even death. Disability is commonly regarded as difficulty performing activities necessary for daily living (Vaughan et al., 2016) that seriously affects elders' quality of life and is associated with institutionalization (Friedman, 2019), increased healthcare costs (Loyalka et al., 2014), and mortality (Dugravot et al., 2020). It can be defined in several ways: the activities of daily living (ADL) and instrumental activities of daily living (IADL) are the most common. The ADL encompasses fundamental activities for independent living at home, which include bathing and feeding oneself. The IADL are more complex, requiring a higher level of autonomy and cognitive function, and necessary for independent life in a community.

Although previous studies have reported relevant links between MCR and several age-related negative outcomes, including depressive symptoms (Xu et al., 2022), dementia (Beauchet et al., 2021), falls (Callisaya et al., 2016), and death (Ayers and Verghese, 2016), very few studies have discussed the associations between MCR and disability. On one hand, previous research has found that subjects with MCI have a high rate of progression to dementia over a relatively short period (Lawton and Brody, 1969), and dementia and MCI shared similar risk factors (Jia et al., 2020). MCR and MCI are two pre-dementia stages with an overlap, which may influence the risk for dementia. On the other, slow gait representing decline in physical function had independent associations with disability onset (Artaud et al., 2015). Additionally, studies also showed that co-occurrence of slow gait and MCI in multiple domains has a higher risk of disability than each condition alone (Doi et al., 2015). Therefore, we hypothesized that older individuals with coexisting cognitive complaints and physical limitations will likely become disabled or lose independence, which was just the association between MCR and disability. Besides, we have found that MCR is a heterogeneous condition with amnestic and non-amnestic subtypes in previous study (Bai et al., 2021). As a heterogeneous condition, MCR has different subtypes based on quantitative gait variables (Allali et al., 2015) or objective cognition measures (Bai et al., 2021), which may have various etiology and pathogenesis. It is essential to investigate the associations between different subtypes of MCR and incidence of disability. Therefore, we used data from the China Health and Retirement Longitudinal Study (CHARLS), a nationally representative prospective study, to further explore the independent association between MCR and its subtype and incident disability among a cohort of community-dwelling older adults.

## Methods

### Study participants

Data for this study originated from the 2011 and 2018 CHARLSs, which surveyed community-dwelling adult populations (aged ≥ 45 years) and their spouses in China. The national baseline survey for CHARLS was conducted between June 2011 and March 2012, and samples were chosen through multi-stage stratified probability proportional to size (PPS) sampling ensuring that the baseline sample closely matched the 2010 census in terms of demographics. The final sample of 150 counties fell within 28 provinces. A total of 17,705 respondents were interviewed at the baseline survey in 2011 and were followed up every 2 years afterward using a face-to-face, computer-assisted personal interview (CAPI) technique. A more detailed description of CHARLS survey design can be found elsewhere (Zhao et al., 2014).

**Figure 1:**
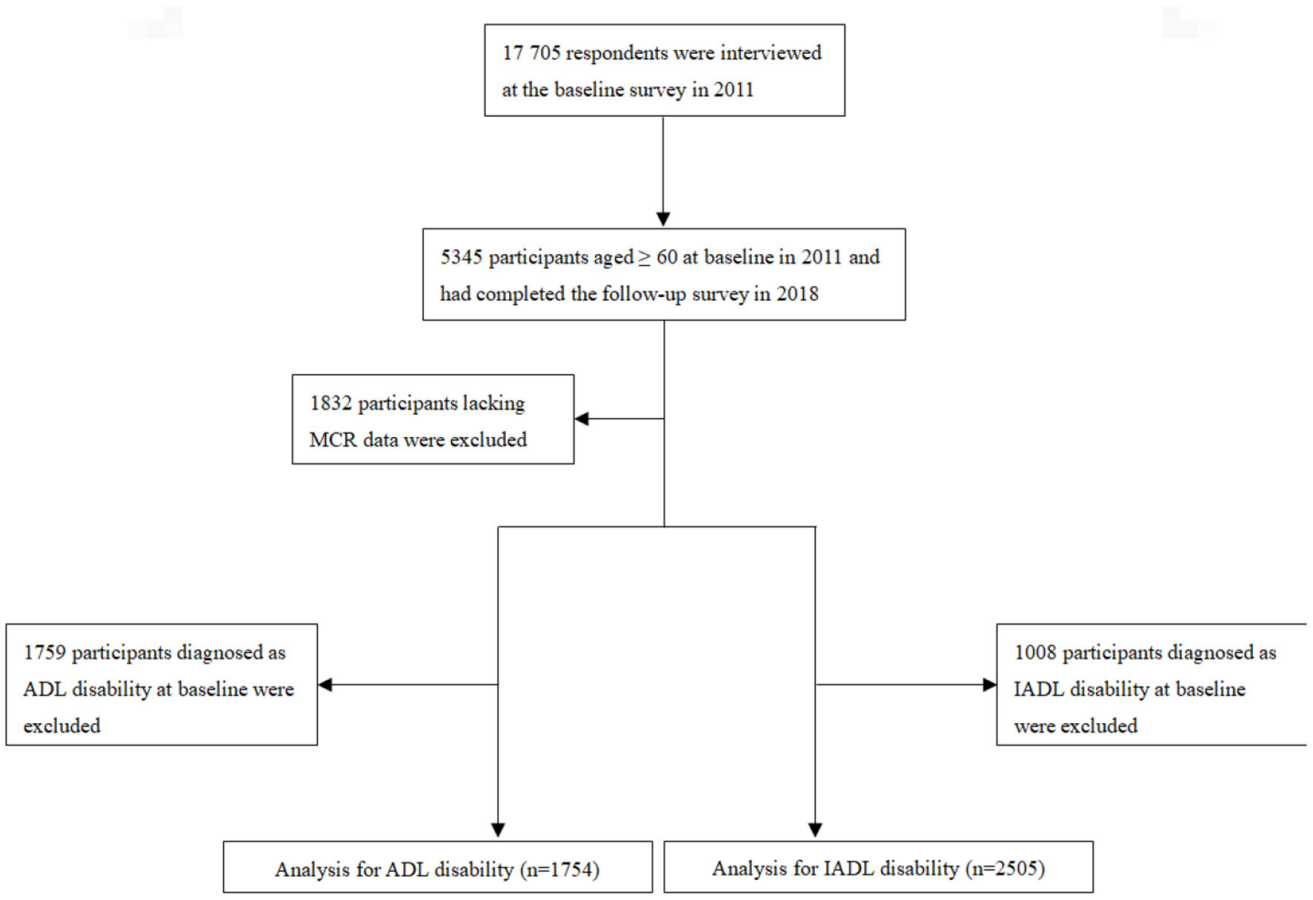
Flowchart of the study identification process.

For this study, we selected an initial sample of 5,345 participants aged ≥ 60 years at the baseline in 2011 who completed the follow-up survey in 2018. ADL and IADL disabilities were measured at both the baseline and the 2018 follow-up survey; subjective cognitive complaints and gait speed were measured only at the baseline. In this study, participants were excluded if they (1) were younger than 60 years old; (2) lacked ADL data in 2011/2018 or had difficulty in ADL at baseline; (3) lacked IADL data in 2011/2018 or had difficulty in IADL at baseline; or (4) lacked MCR data at baseline. A total of 5,345 participants aged ≥ 60 years completed the follow-up survey in 2018: 1,832 participants lacking baseline MCR data were first excluded, and 1,759 participants diagnosed with ADL disability and 1,008 participants diagnosed with IADL disability at baseline were further excluded. Finally, 1,754 participants were included in the analysis for ADL disability, and 2,505 were included in the analysis for IADL disability. All participants provided written informed consent, and the ethics panel of the Ethical Review Committee approved this study. Figure 1 provides more details of the study population inclusion process.

### Assessment of disability

Disability was derived from the participants' reports of any difficulty in ADLs and IADLs and scaled. The ADL and IADL scales are the most commonly used disability assessment measures. The ADL scale used in CHARLS covers dressing, bathing, eating, getting in/out of bed, using the toilet, and controlling urination or defecation (Katz et al., 1963; Xu et al., 2020; Qiao et al., 2021). The ADL scale has four levels of answers for each question: “no difficulty,” “have difficulty but can still do it,” “have difficulty and need help,” and “cannot do it.” The IADL scale includes five items based on Lawton's index (Lawton and Brody, 1969; Xu et al., 2020; Qiao et al., 2021): managing money, taking medication, shopping, meal preparation, and doing housework. The answer items on both the IADL and ADL scales are the same. In subsequent analysis, disability was defined as a dichotomous variable with an assigned value of 1 if the respondents had some difficulty or could not do the activities and 0 otherwise.

### Assessment of MCR

Diagnosis of MCR was based on the original criteria proposed by Verghese et al. (2014), cognitive complaints and slow gait speed without dementia or impaired mobility. In our study, subjective cognitive complaints were assessed using a self-reported question about memory loss: “How would you rate your memory at the present time?” Respondents were asked to rank their answers as excellent, very good, good, fair, or poor. Those who responded “fair” or “poor” were recorded as having cognitive complaints. Furthermore, the average time respondents took to walk along a straight path twice was used to compute gait speed. A walking velocity of 2.5 m was used as an objective measurement of gait speed. The gait speed test was conducted in an open space without carpet approximately 4 m in length, and a 2.5-m line was set up as the walking route. The interviewer would ask the respondents to walk along the route twice and would follow alongside them. When the test started, each respondent would raise their leg and begin walking from the starting point of the test. Walking aids were allowed during the test. We defined slow gait speed as a speed ≤1.0 SD from age- and sex-appropriate mean values of gait speed in this cohort.

In subgroup analysis, subjects were characterized as having MCR-memory impairment (MI) if there was MI or MCR-non-MI if there was no MI (Bai et al., 2021). Episodic memory was evaluated through immediate recall and delayed recall in a word-recall test in CHARLS, and participants were requested to immediately repeat 10 Chinese words after these were read to them by interviewers. Then, participants were again asked to recall as many of the original words as possible after being asked questions concerning their depression status (4–10 min). A successful recall was coded as 1, while an unsuccessful recall was coded as 0. Scores were in the range 0–10 for immediate and delayed recalls. Episodic memory was evaluated using the means of the immediate and delayed word-recall scores. We defined MI as 1.0 SD or more below the mean value of episodic memory in this cohort.

### Covariates

Age, gender, educational level, residential area, and marital status were obtained through a self-reported questionnaire. Education was assessed as the highest level of educational qualification attained, and all participants were divided into three groups: illiterate, primary education or above, and secondary education or above. According to participants' living region, residential area was defined as rural or urban. Marital status was defined as married or unmarried (including divorced, widowed, and never married). Information on chronic diseases, including any history of hypertension, diabetes, coronary heart disease, and stroke was self-reported.

CHARLS questionnaire asked respondents about their participation in 11 social activities in the past month. If any of the options were selected, respondents were considered to have participated in social activities; otherwise, they were considered to not have participated in social activities. We constructed the variable of physical activity (PA) scores based on the International Physical Activity Questionnaire (IPAQ), including activities for exercise, entertainment, job demand, and other purposes. General questions about PA use in CHARLS questionnaire included the amount of time a person spent on different types of PA (vigorous activities, moderate activities, and walking for at least 10 min continuously) in a usual week. According to the responses, we indexed the amount of physical activity in 1 day as 1 (<0.5 h); 2 (0.5–2 h); 3 (2–4 h); and 4 (>4 h). The score was calculated by multiplying the number of days and the daily PA duration index for each activity. Finally, we generated the variable of PA score using metabolic equivalent (MET) multipliers as follows: PA score = 8.0 × total vigorous activity weekly duration score + 4.0 × total moderate activity weekly duration score + 3.3 × total walking weekly duration score (Deng and Paul, 2018). Depressive symptoms were measured using the Center for Epidemiologic Studies Depression Scale (CES-D-10) in the CHARLS, with higher scores indicating more serious symptoms. We used a cut-off score of ≥10 to distinguish participants with depression from those who were relatively free of depression (Andresen et al., 1994).

In addition, we analyzed their current smoking and alcohol consumption habits. All participants were classified into one of two groups of non- or former drinker/smoker and current drinker/smoker.

### Statistical analyses

The Wilcoxon rank-sum test for continuous variables and a chi-squared test for categorical variables were used as appropriate to assess when participants' baseline characteristics did and did not meet the criteria for each MCR subtype. Multiple logistic regression analysis was used to assess the independent effect of MCR at baseline on the odds of ADL/IADL disability at a 7 years follow-up in the sample population after adjustment for socioeconomic status, marital status, urban residence, educational level, baseline self-reported chronic diseases, and current smoking/drinking status. In addition, we repeated the analyses for MCR-MI and MCR-non-MI participants. We reported multivariate ORs from the logistical model along with the 95% confidence interval (CI). All reported *p*-values were two-tailed, with the significance level set at 0.05. All analyses were performed using STATA software (version 14.0; Stata Corp LP, College Station, TX, USA).

## Results

### Characteristics of included participants

Table 1 shows the characteristics of participants included in the longitudinal analysis of ADL disability. None of the participants had difficulty in the ADL at baseline. The prevalence of MCR was 14.4% for all participants, and the prevalence of each subtype was 11.4% for MCR-non-MI and 3.0% for MCR-MI. Moreover, we provided a comparison of participants' characteristics according to MCR subtype and in participants without MCR. The following participants' characteristics were found to be significantly different in each group (*p* < 0.05): mean age (participants with MCR-MI were older compared to those with MCR-non-MI or without MCR), education (a higher proportion of participants with MCR-MI had not received formal education or were illiterate), history of hypertension (a higher proportion of participants with MCR-MI had a history of hypertension), history of stroke (a higher proportion of participants with MCR-MI had a history of stroke), social participation (a lower proportion of participants with MCR-MI had participated in social activities), episodic memory scores (a lower mean scores of participants with MCR-MI than other subgroups) and gait speed (participants with MCR symptoms walked slower than other subgroups, with the lowest mean gait speed among MCR-MI participants).

**Table 1:** Characteristics of study participants for ADL disability analysis (*N* = 1,754).

		**MCR group**			
	**Non-MCR (*N* = 1,502)**	**MCR-non-MI (*N* = 200)**	**MCR-MI (*N* = 52)**	**Total Sample (*N* = 1,754)**	***P*-value**
Age, years (mean ± SD)	66.7 ± 5.79	66.9 ± 5.74	70.1 ± 6.30	67.1 ± 6.12	<0.001
Female, *n* (%)	882 (58.72%)	112 (56.00%)	32 (61.54%)	1,026 (59.49%)	0.181
Urban residence, *n* (%)	492 (32.76%)	78 (39.00%)	16 (30.77%)	586 (33.41%)	0.489
Education, *n* (%)					<0.001
No formal education or illiterate	895 (59.59%)	126 (63.00%)	40 (76.92%)	1,061 (60.49%)	
Primary or above	393 (26.17%)	40 (20.00%)	10 (19.23%)	443 (25.26%)	
Secondary or above	214 (14.25%)	34 (17.00%)	2 (3.85%)	250 (14.25%)	
Current smokers, *n* (%)	421 (28.03%)	64 (32.00%)	11 (21.15%)	496 (28.28%)	0.06
Current drinkers, *n* (%)	387 (25.78%)	51 (25.50%)	14 (27.45%)	452 (25.89%)	0.646
History of hypertension, *n* (%)	499 (33.33%)	71 (35.86%)	22 (42.31%)	592 (33.89%)	0.027
History of diabetes, *n* (%)	110 (7.41%)	9 (4.59%)	5 (9.80%)	124 (7.16%)	0.891
History of CHD, *n* (%)	248 (16.58%)	33 (16.58%)	7 (13.73%)	288 (16.49%)	0.943
History of stroke, *n* (%)	34 (2.27%)	6 (3.00%)	1 (1.92%)	41 (2.34%)	0.033
Depressive symptoms, *n* (%)	715 (47.60%)	93 (46.50%)	32 (61.54%)	840 (47.89%)	0.130
Social Participation, *n* (%)	723 (48.14%)	103 (51.50%)	16 (30.77%)	842 (48.00%)	0.028
Baseline total physical activity score (mean ± SD)	55.48 ± 98.17	39.75 ± 92.82	30.06 ± 72.80	52.94 ± 97.08	1.000
Baseline episodic memory score (mean ± SD)	2.73 ± 1.78	3.09 ± 1.27	0.14 ± 0.23	2.70 ± 1.76	0.000
Baseline gait speed (mean ± SD)	0.67 ± 0.18	0.31 ± 0.08	0.30 ± 0.08	0.61 ± 0.21	0.000

Source: CHARLS 2011.

ADL, activities of daily living; MCR, motoric cognitive risk syndrome; MI, memory impairment; CHD, coronary heart disease.

Table 2 shows the baseline characteristics of participants included in the longitudinal analysis for IADL disability. The prevalence of MCR was 12.8% for all participants, and the prevalence of each subtype was 10.8% for MCR-non-MI and 2.0% for MCR-MI. Similarly, we also compared the participants' characteristics according to MCR subtypes and in participants without MCR. The following participants' characteristics were found to be significantly different in each group (*p* < 0.05): mean age (participants with MCR-MI were older than those with MCR-non-MI or without MCR), education (a higher proportion of participants with MCR-MI had not received formal education or were illiterate), depressive symptoms (a higher proportion of participants with MCR-MI had depressive symptoms), social participation (a lower proportion of participants with MCR-MI had participated in social activities), episodic memory scores (a lower mean scores of participants with MCR-MI than other subgroups) and gait speed (participants with MCR symptoms walked slower than other subgroups, with the lowest mean gait speed among MCR-MI participants).

**Table 2:** Characteristics of study participants for IADL disability analysis (*N* = 2,505).

		**MCR group**			
	**Non-MCR (*N* = 2,185)**	**MCR-non-MI (*N* = 271)**	**MCR-MI (*N* = 49)**	**Total sample (*N* = 2,505)**	***P*-value**
Age, years (mean ± SD)	66.4 ± 5.6	66.5 ± 5.4	69.2 ± 6.2	66.5 ± 5.5	0.015
Female, *n* (%)	1,071 (49.02%)	124 (45.76%)	29 (59.18%)	1,224 (48.86%)	0.206
Urban residence, *n* (%)	754 (34.51%)	104 (38.38%)	16 (32.65%)	874 (34.89%)	0.428
Education, *n* (%)					0.004
No formal education or illiterate	1,176 (53.82%)	134 (49.45%)	37 (75.51%)	1,347 (53.77%)	
Primary or above	623 (28.51%)	74 (27.31%)	9 (18.37%)	706 (28.18%)	
Secondary or above	386 (17.67%)	63 (23.25%)	3 (6.12%)	452 (18.04%)	
Current smokers, *n* (%)	701 (32.08%)	94 (34.69%)	11 (22.45%)	806 (32.18%)	0.156
Current drinkers, *n* (%)	716 (32.77%)	84 (31.00%)	18 (36.73%)	818 (32.65%)	0.912
History of hypertension, *n* (%)	633 (28.97%)	82 (30.26%)	19 (38.78%)	734 (29.30%)	0.307
History of diabetes, *n* (%)	125 (5.72%)	13 (4.80%)	3 (6.12%)	141 (5.63%)	0.815
History of CHD, *n* (%)	297 (13.59%)	39 (14.39%)	5 (10.20%)	341 (13.61%)	0.732
History of stroke, *n* (%)	37 (1.69%)	2 (0.74%)	1 (2.04%)	40 (1.60%)	0.481
Depressive symptoms, *n* (%)	885 (40.50%)	110 (40.59%)	23 (46.94%)	1,018 (40.64%)	0.004
Social Participation, *n* (%)	1,102 (50.43%)	134 (49.45%)	12 (24.49%)	1,248 (49.82%)	0.012
Baseline total physical activity score (mean ± SD)	59.36 ± 103.62	56.23 ± 104.72	32.72 ± 82.02	58.51 ± 103.39	1.000
Baseline episodic memory score (mean ± SD)	2.94 ± 1.77	3.41 ± 1.43	0.11 ± 0.21	2.93 ± 1.77	0.000
Baseline gait speed (mean ± SD)	0.69 ± 0.19	0.32 ± 0.08	0.30 ± 0.08	0.64 ± 0.22	0.000

Source: CHARLS 2011.

IADL, instrumental activities of daily living; MCR, motoric cognitive risk syndrome; MI, memory impairment; CHD, coronary heart disease.

### Longitudinal association between MCR and ADL/IADL disability

Tables 3, 4 show longitudinal associations between MCR and incident disability. Among the 1,754 subjects who did not have ADL disability at baseline, 608 (34.66%) developed incident ADL disability during the 7 years prior to follow-up; among the 2,538 subjects who did not have IADL disability at baseline, 803 (31.64%) had developed incident IADL disability by the 7 year follow-up.

**Table 3:** Multiple logistic regressions presenting the longitudinal association of MCR with ADL disability and IADL disability.

	ADL Disability (*N* = 1,754)	
MCR group	All population	
	OR (95% CI)	*P*-value
Non-MCR	1.00	
MCR	1.57 (1.19–2.09)^a^	0.002
	IADL disability (*N* = 2,505)	
MCR group	All population	
	OR (95% CI)	*P*-value
Non-MCR	1.00	
MCR	1.46 (1.13–1.88)^a^	0.004

MCR, motoric cognitive risk syndrome; ADL, activities of daily living; IADL, instrumental activities of daily living; OR, odds ratio; CI, confidence interval.

a sAdjusted for age, gender, marital status, urban residence, educational level, baseline self-reported chronic diseases, social participation, depressive symptoms, baseline episodic memory scores, and current smoking/drinking status.

**Table 4:** Multiple logistic regressions presenting the longitudinal association of MCR subtypes with ADL disability and IADL disability.

	ADL disability (*n* = 1,754)	
MCR group	All population	
	OR (95% CI)	*P*-value
Non-MCR	1.00	
MCR-non-MI	1.57 (1.15–2.14)^a^	0.005
MCR-MI	1.66 (0.92–2.99)^a^	0.092
	IADL disability (*n* = 2,505)	
MCR group	All population	
	OR (95% CI)	*P-*value
Non-MCR	1.00	
MCR-non-MI	1.35 (1.02–1.78)^a^	0.035
MCR-MI	2.14 (1.18–3.88)^a^	0.012

MCR, motoric cognitive risk syndrome; ADL, activities of daily living; IADL, instrumental activities of daily living; OR, odds ratio; CI, confidence interval; MI, memory impairment.

a Adjusted for age, gender, marital status, urban residence, educational level, baseline self-reported chronic diseases, social participation, depressive symptoms, baseline episodic memory scores, and current smoking/drinking status.

We found that MCR was significantly associated with an increased risk of ADL disability in the total population. MCR at baseline was associated with 58% increased odds of incident ADL disability (OR = 1.58, 95% CI: 1.19–2.09) and 46% increased odds of incident IADL disability (OR = 1.46, 95% CI: 1.13–1.88). We further analyzed the associations between subtypes of MCR with incident ADL/IADL disability and found that compared with healthy controls, participants with MCR-non-MI were associated with a 52.7% increased risk of ADL disability and with a 35.8% increased risk of IADL disability. Participants with MCR-MI were not significantly associated incident ADL disability, while associated with even higher incidence of IADL disability (OR = 2.14, 95% CI: 1.18–3.88) compared with healthy controls.

## Discussion

In this large, nationally representative sample of older Chinese adults, our study provided strong evidence that MCR could predict incident ADL/IADL disability in a non-disabled population. In addition, we found that MCR-MI was associated with a higher risk of future IADL disability than MCR-non-MI. Our results indicated that MCR could be used as a useful screening tool to identify individuals at high risk of incident disability.

### Prevalence of ADL/IADL

The disability prevalence on ADL/IADL shows associations with aging (Ward et al., 1995), gender, chronic diseases (Fried and Guralnik, 1997), economy, exercise, and the use of medications (Dunlop et al., 1997). In this study, higher prevalence of ADL (34.66%) was reported compared with the prevalence of IADL (31.64%) in a 7 year period, which is not, however, consistent with existing evidence from China: Liu et al. (2020). presented that prevalence of ADL disability decreased from 58% in 2011 to 56% in 2015, with prevalence of IADL increasing from 24 to 29% for the same periods using CHARLS data, and Tang et al. (1999). suggested that the functional disability prevalence was 6.5% on ADL and 7.9% on IADL among urban, plain rural and mountain rural regions of Beijing. By contrast, Feng et al. (2013). found that the trend toward improvement in IADL function is more consistent and substantial than that of ADL function from 1998 to 2008 among elder adults in Shanghai, China. This is probably due to the inclusion of relatively higher percentage (65%) of participants living in rural areas, as compared with Feng's et al. (2013) (30%) and Tang's et al. (1999) study (33%). Also, more than 90% of the respondents were from rural area in Liu's et al. (2020) study. It could also be the results of different ADL/IADL items used in these studies (Allain et al., 1997). For the convenience of future comparisons between different economic groups, more unified inclusion criterias and functional tasks need to be devised and implemented.

### Associations between MCR syndrome, MCR subtypes, and disability

Our findings concur with the results of the few prior studies to examine the relationship between MCR and disability and supplement these studies' results. Previous studies showed that declines in physical (Abe et al., 2019; Nanjo et al., 2021) and cognitive function (Dotchin et al., 2015; Basile and Sardella, 2021) were independent risk factors of disability, and only Doi et al. (2017) found that MCR predicted risk for disability (operationally defined as receiving LTCI certification) among 4,235 Japanese community-dwelling older adults (HR = 1.69, 95% CI 1.08–2.02, *p* < 0.001). They also reported that the co-occurrence of MCI and slow gait was related to a higher risk of disability compared with MCI or slow gait in another study (Doi et al., 2015). However, no previous research was conducted on the association between MCR subtypes and incident disability. Furthermore, in the subgroup analysis, we found that MCR-MI was associated with higher odds of IADL disability compared with MCR-non-MI, which indicated that memory impairment might predict future physical disability; this confirms the findings of other population-based cohorts. Yaffe et al. (2009) found that maintenance of cognitive function over the first 4 years of follow-up was associated with a lower risk of mortality and incident disability over the subsequent 3 years of follow-up among 2,733 older adults living in Memphis, TN or Pittsburgh, PA, and Johnson et al. (2007) demonstrated that individuals with poor executive function—either with or without impaired mMMSE scores—were more likely to have prevalent functional difficulty when compared with women without cognitive impairment.

Recently, research efforts have focused on MCR subtypes. Our previous study (Bai et al., 2021) among community-dwelling older Chinese adults found that higher C-reactive protein (CRP) levels were significantly associated with higher odds of MCR and MCR-MI, while no relationship was found between CRP and the odds of MCR-non-MI. Allali et al. (2016) conducted research among 314 community-residing older adults in New York and defined four new subtypes of MCR by substituting slow gait with short stride length (MCRsl), slow swing time (MCRsw), high stride length variability (MCRslv), and high swing time variability (MCRswv), reporting that MCR subtypes based on individual gait parameters show commonalities and differences in cognitive profiles and risk factors. Future studies should define MCR subtypes based on both cognition and walking parameters to comprehensively explore the potential mechanisms of MCR, as well as the relationship between MCR and multiple adverse outcomes, such as disability.

### Potential mechanisms

Maintaining a normal, well-balanced gait requires the efficient integration of motoric, cognitive, and psychological functions; therefore, the inability to maintain a normal gait could result in disability. Besides, impairments in cognitive domains such as executive function, attention, processing speed, and memory are known to increase the risk of disability. Since both physical and cognitive impairments increased the odds of an individual becoming disabled, it is plausible that their combination would exacerbate the decline in functional ability among elders. A previous study (Heiland et al., 2019) also found that physical inactivity was a risk factor in physical and cognitive impairment, and it was associated with incident ADL disability (HR = 1.99, 95% CI 1.36–2.93). Whether physical inactivity mediates between MCR and disability remains to be discussed.

### Clinical implications

Our findings add value to the current body of evidence showing that MCR is a significant predictor of disability, which has policy implications for health and social service needs. A longitudinal survey (Ansah et al., 2021) in China found that 21.8% of older Chinese adults will develop impairment (functional disability or cognitive impairment) by 2060, which highlights the importance of early identification of older adults at high risk of disability.

Studies have shown that gait speed is a very strong predictor of disability (Artaud et al., 2015), and cognitive impairment also predict disability (Dodge et al., 2005). MCR involves both cognition and gait and could therefore be a more sensitive predictor of disability. As slow gait may capture other non-cognitive pathways leading to disability, the combination of both slow gait and cognitive impairment has expanded the clinical utility of MCR in predicting non-dementia outcomes, such as disability and mortality. Since the diagnosis of MCR does not require neuropsychological tests, and gait speed is commonly used in health assessments among older adults without requiring elaborate equipment, it is feasible and useful to apply MCR in future early detection and preventive strategies to help avoid disability.

### Strengths and limitations

To our knowledge, this study is the first to examine the role of MCR and its subtypes as risk factors for incident ADL/IADL disability among community-dwelling older adults. For primary healthcare and public health practitioners, this information indicates that attention should be paid to individuals with MCR-MI or MCR-non-MI. The discrepancy between MCR subtypes regarding episodic memory function should also be emphasized in clinical settings. Moreover, since a reliable measurement of outcomes has been made, and a comprehensive assessment of the sociodemographic and clinical characteristics associated with ADL or IADL disability has been completed and adjusted, our study provided useful information for informing effective interventions and improving both the mental and physical health of Chinese elders. However, some noteworthy limitations remain. First, the characterization of MCR subtypes was based on answers to questionnaires without the support of a psychiatric diagnosis, which could result in inaccurate diagnoses because of memory disorders among older individuals. Second, although we controlled for many important factors of disability, the possibility of unmeasured confounders cannot be excluded. Finally, there may be selection bias regarding our longitudinal sample, given that individuals who did not complete the survey in either of the two waves were removed from the study. The respondents who remained in our analyses might have been healthier than those who were excluded.

## Conclusions

In summary, our study demonstrated that MCR and its subtypes are independently associated with an increased risk of both incident ADL and IADL disabilities.

Prevention of risk factors of cognitive impairment and slow gait may decrease the potential risk of MCR and in turn reduce the risk of ADL/IADL disability. Moreover, special attention should be given to the care of older adults with MCR-MI. Effective interventions, such as physical exercise and social participation, should be planned to reduce the prevalence of disability among older individuals.

## Data availability statement

Publicly available datasets were analyzed in this study. This data can be found at: http://charls.pku.edu.cn/en.

## Ethics statement

The studies involving human participants were reviewed and approved by Peking University's institutional review board. The patients/participants provided their written informed consent to participate in this study.

## Author contributions

AB, WX, and ZL: study concept, design, and constructive discussions. AB, WB, and HJ: analysis and interpretation of data. AB, WB, HJ, and WX: preparation of manuscript. All authors read and approved the final manuscript.

## Funding

This study was supported by National Key R&D Program of China (2018YFC2000301).

## Conflict of interest

The authors declare that the research was conducted in the absence of any commercial or financial relationships that could be construed as a potential conflict of interest.

## Publisher's note

All claims expressed in this article are solely those of the authors and do not necessarily represent those of their affiliated organizations, or those of the publisher, the editors and the reviewers. Any product that may be evaluated in this article, or claim that may be made by its manufacturer, is not guaranteed or endorsed by the publisher.
